# HRMS Characterization, Antioxidant and Cytotoxic Activities of Polyphenols in *Malus domestica* Cultivars from Costa Rica

**DOI:** 10.3390/molecules26237367

**Published:** 2021-12-04

**Authors:** Mirtha Navarro-Hoyos, Elizabeth Arnáez-Serrano, Silvia Quesada-Mora, Gabriela Azofeifa-Cordero, Krissia Wilhelm-Romero, Maria Isabel Quirós-Fallas, Diego Alvarado-Corella, Felipe Vargas-Huertas, Andrés Sánchez-Kopper

**Affiliations:** 1 Bioactivity & Sustainable Development (BIODESS) Group, Department of Chemistry, University of Costa Rica (UCR), San Jose 2060, Costa Rica; krissia.wilhelm@ucr.ac.cr (K.W.-R.); maria.quirosfallas@ucr.ac.cr (M.I.Q.-F.); luis.alvaradocorella@ucr.ac.cr (D.A.-C.); luis.vargashuertas@ucr.ac.cr (F.V.-H.); 2 Department of Biology, Costa Rica Institute of Technology (TEC), Cartago 7050, Costa Rica; earnaez@itcr.ac.cr; 3 Department of Biochemistry, School of Medicine, University of Costa Rica (UCR), San Jose 2060, Costa Rica; silvia.quesada@ucr.ac.cr (S.Q.-M.); gabriela.azofeifacordero@ucr.ac.cr (G.A.-C.); 4 CEQIATEC, Department of Chemistry, Costa Rica Institute of Technology (TEC), Cartago 7050, Costa Rica; ansanchez@itcr.ac.cr

**Keywords:** *Malus domestica*, apple, UPLC, ESI-MS, mass spectrometry, polyphenols, flavonoids, procyanidins, nutraceutic, antioxidant, antitumoral

## Abstract

There is increasing interest in research into fruits as sources of secondary metabolites because of their potential bioactivities. In this study, the phenolic profiles of *Malus domestica* Anna and Jonagold cultivars from Costa Rica were determined by Ultra Performance Liquid Chromatography coupled with High Resolution Mass Spectrometry (HRMS) using a quadrupole-time-of-flight analyzer (UPLC-QTOF-ESI MS), on enriched-phenolic extracts from skins and flesh, obtained through Pressurized Liquid Extraction (PLE). In total, 48 different phenolic compounds were identified in the skin and flesh extracts, comprising 17 flavan-3-ols, 12 flavonoids, 4 chalcones, 1 glycosylated isoprenoid and 14 hydroxycinnamic acids and derivatives. Among extracts, the flesh of Jonagold exhibits a larger number of polyphenols and is especially rich in procyanidin trimers, tetramers and pentamers. Evaluating total phenolic content (TPC) and antioxidant activities using ORAC and DPPH procedures yields higher values for this extract (608.8 mg GAE/g extract; 14.80 mmol TE/g extract and IC_50_ = 3.96 µg/mL, respectively). In addition, cytotoxicity evaluated against SW620 colon cancer cell lines and AGS gastric cancer cell lines also delivered better effects for Jonagold flesh (IC_50_ = 62.4 and 60.0 µg/mL, respectively). In addition, a significant negative correlation (*p* < 0.05) was found between TPC and cytotoxicity values against SW620 and AGS adenocarcinoma (*r* = −0.908, and −0.902, respectively). Furthermore, a significant negative correlation (*p* < 0.05) was also found between the number of procyanidins and both antioxidant activities and cytotoxicity towards SW620 (*r* = −0.978) and AGS (*r* = −0.894) cell lines. These results align with Jonagold flesh exhibiting the highest abundance in procyanidin oligomers and yielding better cytotoxic and antioxidant results. In sum, our findings suggest the need for further studies on these Costa Rican apple extracts—and particularly on the extracts from Jonagold flesh—to increase the knowledge on their potential benefits for health.

## 1. Introduction

The increasing popularity and acceptability of herbal medicine is based on natural products being safe and readily available [1]. This awareness is well justified, since evidence of the past decades demonstrates the medicinal properties and functionalities of dietary derived natural compounds and their several health implications. Thus, people who consume higher amounts of fruits and vegetables have better outcomes in terms of prevention of heart disease, cancer and autoimmune diseases, but also as a protective layer against asthma, cataracts, diabetes, Alzheimer, among others diseases [2].

Oxidative stress is a central mechanism of disease and aging. While reactive oxygen species (ROS) are produced in normal aerobic metabolism, the imbalance of oxidative homeostasis is responsible for the further disruption of biomolecules such as lipids, proteins, DNA and carbohydrates, and thus alters their biological functions as signaling cascades and structural capacity [3]. The human body has complex antioxidant defense mechanisms, but these can fail, leading to the accumulation of ROS. There is sufficient evidence suggesting that an increase in the production of ROS can contribute to developing chronic diseases such as neurodegeneratives [4], cancer [5], cardiovascular [6] and infectious diseases [7].

Antioxidant compounds such as dietary polyphenols can counteract the effect of this oxidative stress through mechanisms of action including different pathways such as direct ROS scavenging, inhibition of enzymes or trace elements chelation, which are involved in free radical generation, and by increasing endogenous antioxidant production [8].

Functional foods are those containing physiologically active components that contribute to health management exerting their effects mainly through antioxidant mechanisms [9], which are at the base of an increasing trend in the consumption of and studies on produce, including legumes and fruits. For instance, apples are a large contributor of the total amount of dietary polyphenols consumed worldwide, representing the largest source of phenolics in the United States and Europe, with 22% of the phenolic consumption from fruits [10].

Polyphenols found in apples, such as flavonoids, have been found to exhibit antioxidant, anticancer, antibacterial, anti-inflammation activities as well as to exert cardioprotective and immune modulator effects [11]. In turn, proanthocyanidins are known to display effective antimicrobial, anticancer, antiproliferative and antiangiogenic activity, and are antihypertensive, anti-obesity, neuroprotective and antiaging agents [12]. In addition, dihydrochalcones have shown to possess cardioprotective, anti-cancer, anti-obesity, anti-diabetic, antioxidant, anti-ageing, hyperglycemia, anti-microbial and anti-inflammatory activities [13,14].

However, despite increased research efforts, existing information is insufficient for most of the dietary sources of polyphenols, hence the growing trend in the consumption of dietary supplements derived from these compounds, which increases the need for accurate and up-to-date information about their chemical and bioactive properties. A significant number of publications are available that indicate that apples have antioxidant activity, and for instance that they inhibit the growth of cancer cells among other health benefits, and many of them attribute these effects to their polyphenols [13,15,16].

Different studies have presented evidence for the diversity of chemical components based on locations and cultivars [13,16,17,18]. Differences in phytochemical composition between apple cultivars are influenced by biotic interactions and show that important characteristics such as microbiome composition are dependent on geographical location and local environment [19,20]. In Costa Rica, Ana and Jonagold apple cultivars were introduced as an initiative of local producers to diversify their crops and to respond to local consumer trends in the country [21]. This explains the interest in establishing chemical composition and bioactivities from these local apple cultivars.

Hence, the objective of the present study is to expand preliminary findings on one Costa Rican cultivar [22], in order to evaluate the anti-cancer effects of new characterized apple cultivars, assessing cytotoxic activity on SW620 colon cancer cells and AGS gastric cancer cells and their antioxidant activity using two methods, oxygen radical absorbance capacity (ORAC) and 2,2-diphenyl-1-picrylhidrazyl (DPPH). Characterization of the polyphenolic profile is achieved through Ultra Performance Liquid Chromatography coupled with High Resolution Mass Spectrometry (UPLC-QTOF-ESI MS) and total polyphenolic contents (TPC) are assessed. Finally, correlation studies were performed with the data obtained.

## 2. Results and Discussion

### 2.1. Phenolic Yield and Total Phenolic Content

The Pressurized Liquid Extraction (PLE) process was applied to the fruit samples as described in the Materials and Methods section to obtain phenolic enriched extracts. Table 1 summarizes these results and shows that the skins of the Jonagold cultivar displayed the highest yield (2.09%), while Anna flesh yielded the lowest result (1.07%). For both apple varieties, skin extracts show higher yields than flesh. The total phenolic contents (TPC) indicate that Jonagold’s flesh shows the highest TPC value (608 gallic acid equivalents (GAE)/g dry extract); significantly higher than the other samples, with Anna flesh showing the lowest value (354.46 mg GAE/g dry extract).

Reports from the literature indicate variability among findings in different apple cultivars with total phenolic contents (TPC) values ranging between 5.2–18.0 mg GAE/g DW for skin and 1.3–3.6 mg GAE/g DW for flesh [23,24] for cultivars from Denmark and Germany. Other studies indicate values between 78.2–201.2 mg GAE/100 g FW for skin and 15.9–109.5 mg GAE/100 g FW for flesh [25,26] for cultivars from Canada and China. Comparing with these findings, our results for skin (7.8–8.7 mg/g DW and 173.6–178.5 mg/100 g FW) are within those ranges while they are higher in the case of flesh (3.8–8.6 mg/g DW and 60.3–121.1 mg/100 g FW).

### 2.2. Profile by UPLC-QTOF-ESI MS Analysis

**Figure 1:**
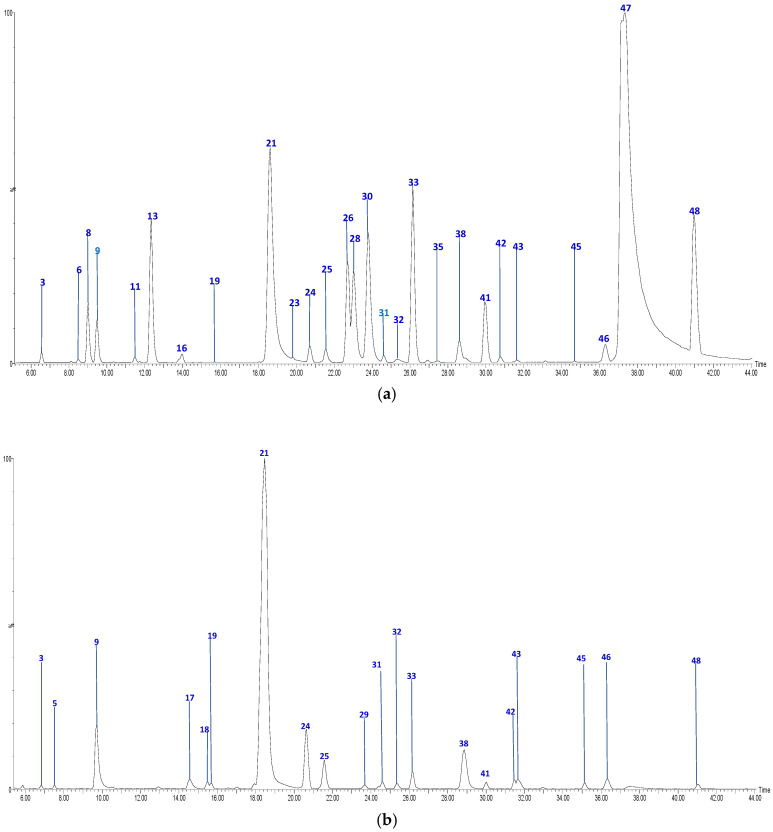
HPLC Chromatograms of *M. domestica* extracts: (**a**) Anna skins (**b**) Anna flesh, in a Phenomenex Luna RP18 C-18 column (150 mm × 4.6 mm × 4 µm) using a Xevo G2-XS QTOF Mass spectrometer (Waters™, Wimslow, UK) in a mass range from 100 to 1500 amu.

**Figure 2:**
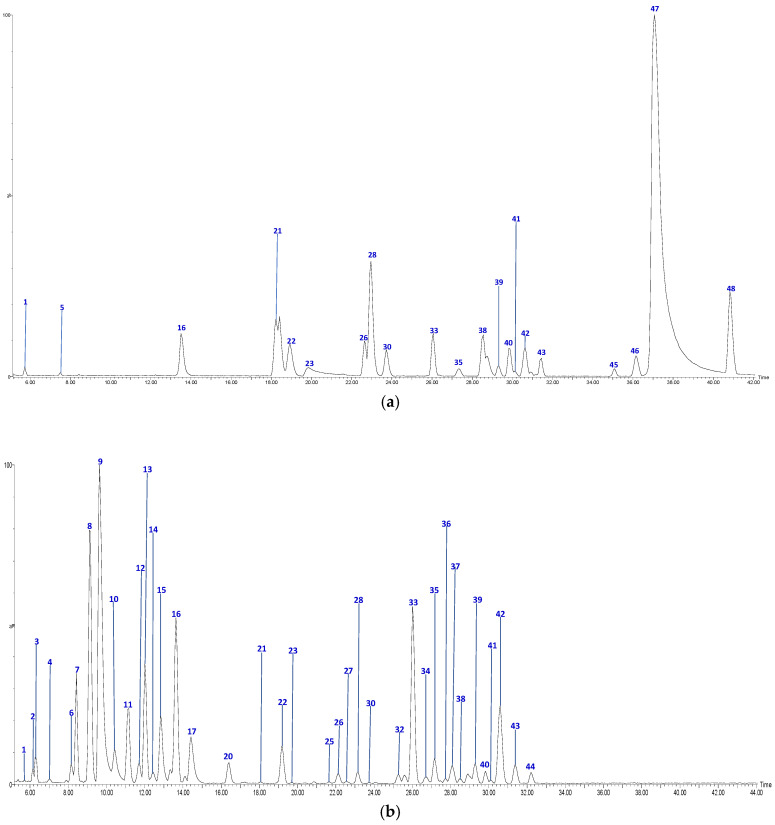
HPLC Chromatograms of *M. domestica* extracts: (**a**) Jonagold skins (**b**) Jonagold flesh, in a Phenomenex Luna RP18 C-18 column (150 mm × 4.6 mm × 4 µm) using a Xevo G2-XS QTOF Mass spectrometer (Waters™, Wilmslow, UK) in a mass range from 100 to 1500 amu.

The UPLC-QTOF-ESI MS analysis described in the Materials and Methods section enabled us to identify 48 different compounds, including 4 chalcones, 15 procyanidin oligomers and the 2 flavan-3-ol monomers, 12 flavonols and glycosylated flavonols, 1 glycosylated isoprenoid derivative and 14 hydroxycinnamic acids and related derivatives (HCA), present in Anna and Jonagold Costa Rican apple cultivars. Figure 1 and Figure 2 show the chromatograms of the four samples and Table 2 summarizes the analysis results for the 48 compounds.

**Figure 3:**
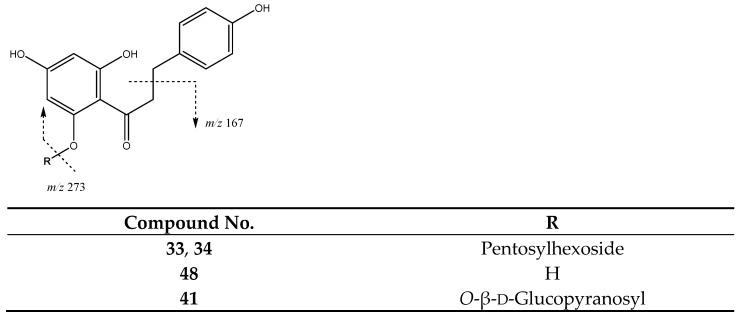
Chalcones structure and main fragments.

Chalcones constitute one group of compounds found in these fruit samples. For instance, peaks **33** (Rt = 26.14 min) and **34** (Rt = 26.95 min) with [M-H]^−^ at *m*/*z* 567.1725 (C_26_H_31_O_14_) were identified as diglycoside derivatives of phloretin. They exhibit a main fragment at *m*/*z* 273 due to the loss of glycosides to yield the phloretin ion aglycone, thus these peaks are tentatively assigned to phloretin-pentosylhexoside isomers (Figure 3). In turn, peak **41** (Rt = 30.00 min) with [M-H]^−^ at *m*/*z* 435.1312 (C_21_H_23_O_10_) is tentatively identified as phloridzin (phloretin 2′-O-glucose) with main fragments at 273 due to the cleavage of the glycoside [27] and at m/z 167 due to loss of the benzylic group. Finally, peak **48** (Rt = 40.98 min) with [M-H]^−^ at *m*/*z* 273.0757 (C_15_H_13_O_5_) is tentatively assigned to the aglycone phloretin, which shows a characteristic main fragment at *m*/*z* 167 due to the loss of the benzylic moiety, as previously described [28,29].

**Figure 4:**
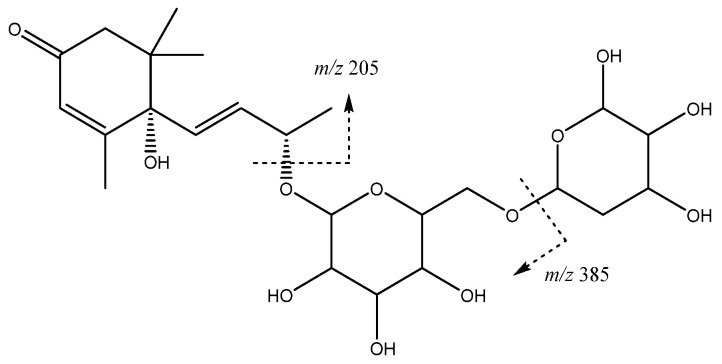
Vomifoliol-pentosylhexoside structure and main fragments.

Another type of compound previously reported in apple is the glycosylated isoprenoid derivatives. In our study, peak **16** (Rt = 13.97 min) with an [M-H]^−^ at *m*/*z* 517.2293 (C_24_H_37_O_12_) was tentatively identified as such, with main fragments at *m*/*z* 385 [M-H-132]^−^ and 205 [M-H-312]^−^ corresponding to the loss of a pentoside and a pentosylhexoside (Figure 4). The resulting ion is coincident with vomifoliol, thus allowing the peak to be assigned to a vomifoliol-pentosylhexoside isomer [30].

**Figure 5:**
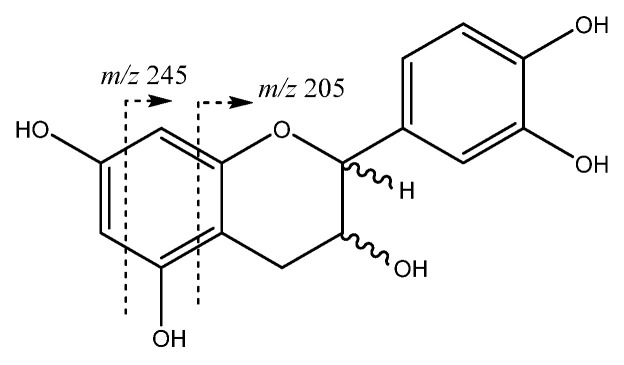
Flavan-3-ols monomers structure and main fragments.

A relevant and abundant group of compounds found in Costa Rican Anna and Jonagold apples is constituted by flavan-3-ols, corresponding to monomers and procyanidin oligomers, including dimers, trimers, tetramers and several pentamers. Firstly, monomers catechin and epicatechin which were present in peaks **8** (Rt = 9.01 min) and **13** (Rt = 12.14 min). As shown in Figure 5, both compounds showed a [M-H]^−^ at *m*/*z* 289.0708 (C_15_H_13_O_6_) with main fragments at *m*/*z* 245 [M-42-H]^−^ due to retro-Diels-Alder fission (RDA) of ring A, *m*/*z* 205 produced by fission of ring A, and *m*/*z* 271 due to loss of water [31].

**Figure 6:**
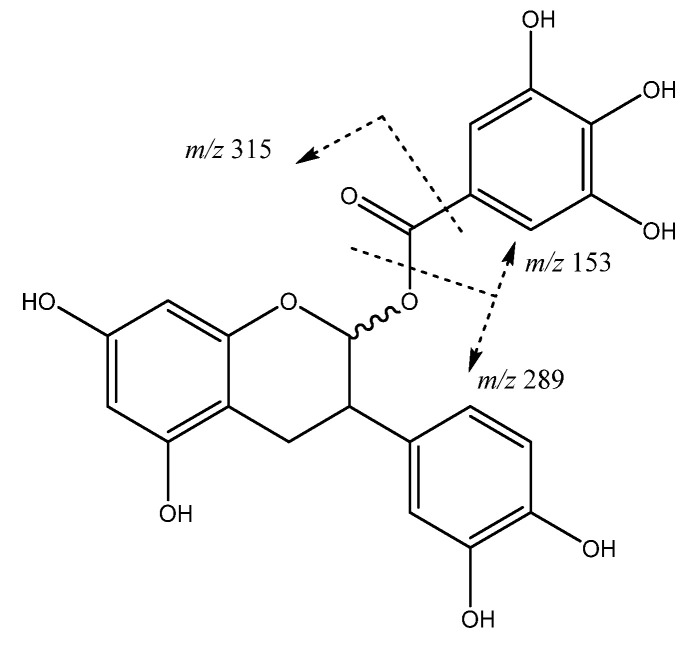
(Epi)-Catechin 3-*O*-gallate structure and main fragments.

In addition, an (epi)-catechin 3-*O*-gallate was present in peak **27** (Rt = 22.89 min) with [M-H] at *m*/*z* 609.1459 (Figure 6), with main fragments at *m*/*z* 289 due to loss of gallate moiety, *m*/*z* 315 and *m*/*z* 153 by α-cleavage of carbonyl group [31].

**Figure 7:**
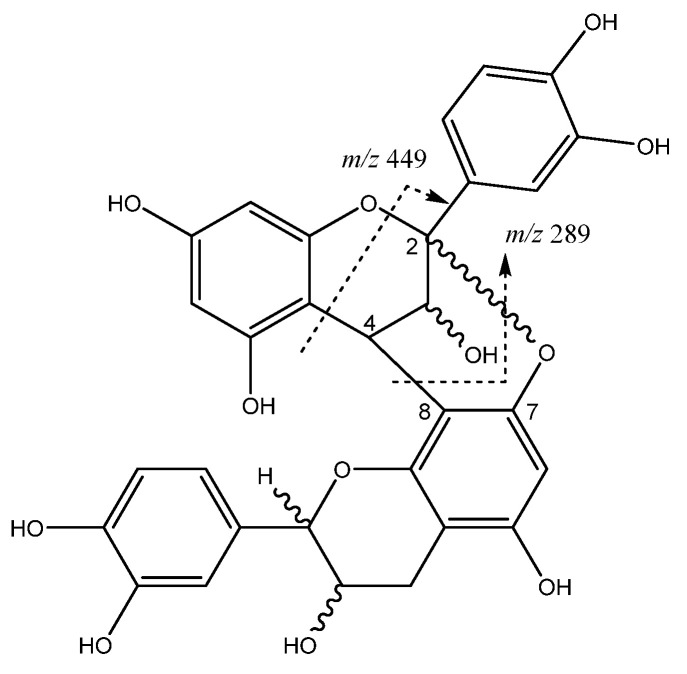
Procyanidin A-type dimer structure and main fragments.

Procyanidin oligomers of monomeric (epi)catechin units can be linked to each other by an interflavonoid C4-C8 link (Figure 7). In the case of procyanidins type A, a linkage in C2-O-C7 is also present [32]. For instance, peak **5** (Rt = 7.67 min) shows a [M-H]^−^ at *m*/*z* 575.1185 (C_30_H_23_O_12_) with a main fragment ion at *m*/*z* 449 [M-H-126]^−^ due to the elimination of a phloroglucinol molecule from this A-type dimer, and a fragment of *m*/*z* 289 that corresponds to the monomer [33].

**Figure 8:**
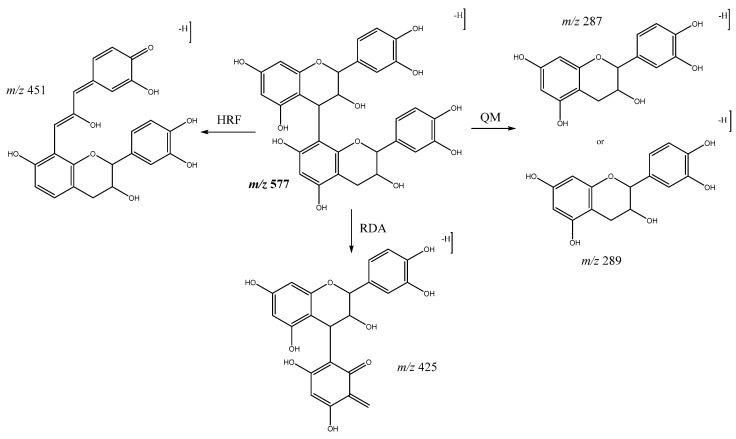
Fragmentation pathways of B-type procyanidin dimers: HRF, Heterocyclic ring fusion; RDA, retro-Diels–Alder; QM, quinone methide.

In our study, procyanidin B-type oligomers linked through a C4-C8 single bond are the most abundant group of flavan-3-ols obtained. Among these, procyanidin B dimers are present in peaks **1** (Rt = 5.74 min), **2** (Rt = 6.12 min), **3** (Rt = 6.57 min), and **9** (Rt = 9.52 min) with [M-H]^−^ at *m*/*z* 577.1343 (C_30_H_25_O_12_). The main fragments shown by these compounds (Figure 8) were at *m*/*z* 559 which originates from water loss and *m*/*z* 451 which is a result of the elimination of the phloroglucinol through heterocyclic ring fission (HRF). As well as *m*/*z* 425 [M-H-152]^−^ and 407 [M-H-170]^−^ from retro Diels-Alder (RDA), the ion at *m*/*z* 289 originates from quinone-methide cleavage (QM) resulting in the ion of the monomer [34].

**Figure 9:**
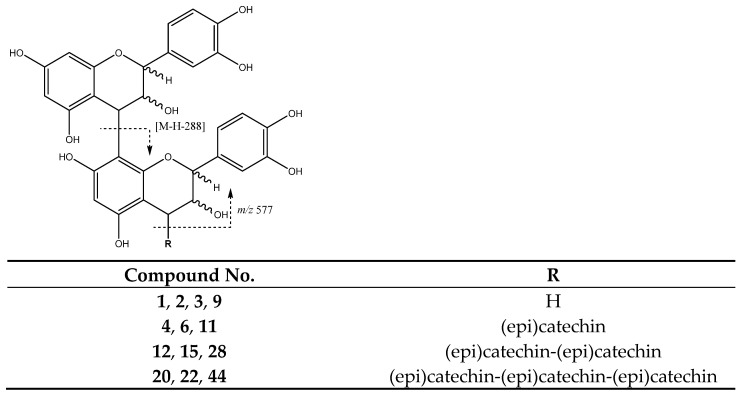
Proanthocyanidin B-type trimers, tetramers and pentamers structures and main fragments.

On the other hand, procyanidin B-type trimers (Figure 9) are shown in peaks **4** (Rt = 7.31 min), **6** (Rt = 8.50 min) and **11** (Rt = 11.48 min) with [M-H]^−^ at *m*/*z* 865.2004 (C_45_H_37_O_18_). They undergo QM cleavage of the upper interflavanoid bond producing ions of *m*/*z* 287 and 577, whereas cleavage of the lower interflavanoid bond forms ions of *m*/*z* 289 and 575 [33].

In addition, as also shown in Figure 9, peaks **12** (Rt = 11.76 min), **15** (Rt = 12.82 min) and **28** (Rt = 23.20 min) with [M-H]^−^ at *m*/*z* 1153.2579 (C_60_H_49_O_24_) correspond to B-type tetramers. Finally, peaks **20** (Rt = 16.55 min), **22** (Rt = 19.13 min) and **44** (Rt = 32.05 min) with [M-H]^−^ at *m*/*z* 1441.2936 (C_75_H_61_O_30_) were tentatively assigned to B-type pentamers. For these compounds, main fragment ions were observed from QM cleavage as multiples of the monomer: *m*/*z* 289, 577 and 865 for tetramers, and additionally *m*/*z* 1153 for pentamers [32].

**Figure 10:**
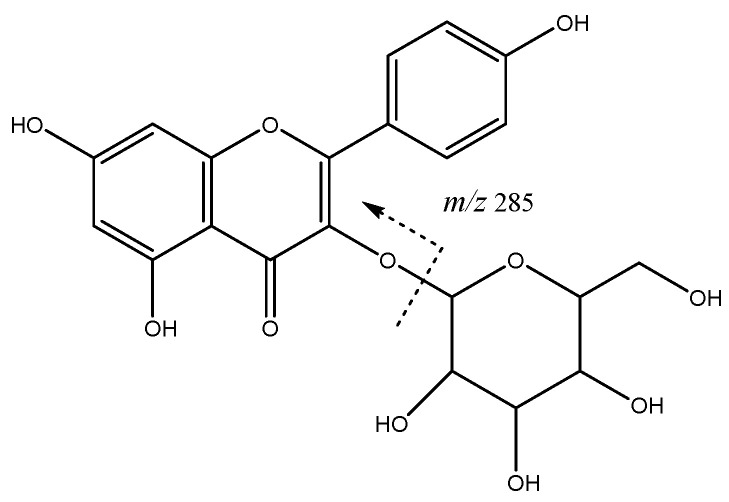
Kaempferol hexoside fragmentation.

Flavonoids constitute another group of compounds found on these apple extracts. For instance, peaks **38** (Rt = 28.63 min) and **46** (Rt = 31.25 min) with [M-H]^-^ at *m*/*z* 447.0928 (C_21_H_19_O_11_) were tentatively assigned to kaempferol hexosides with main fragment at *m*/*z* 285 (Figure 10).

**Figure 11:**
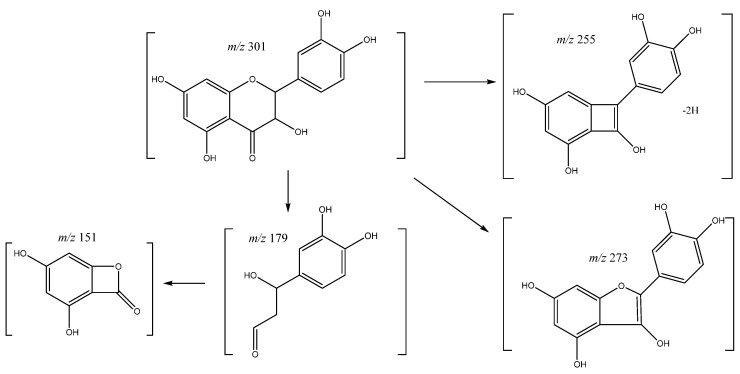
Quercetin fragmentation pathway.

On the other hand, quercetin was assigned to peak **47** (Rt = 37.25) showing a negative molecular ion [M-H]^−^ at *m*/*z* 301.0353 (C_15_H_9_O_7_). The main fragment ions (Figure 11) were found at *m*/*z* 179 and 151 from retrocyclization pathway [35], *m*/*z* 283 [M-18-H]^−^ due to loss of water, *m*/*z* 273 [M-28-H]^−^ from the loss of CO, and *m*/*z* 255 [M-18-28-H]^−^ due to loss of water and CO [36].

**Figure 12:**
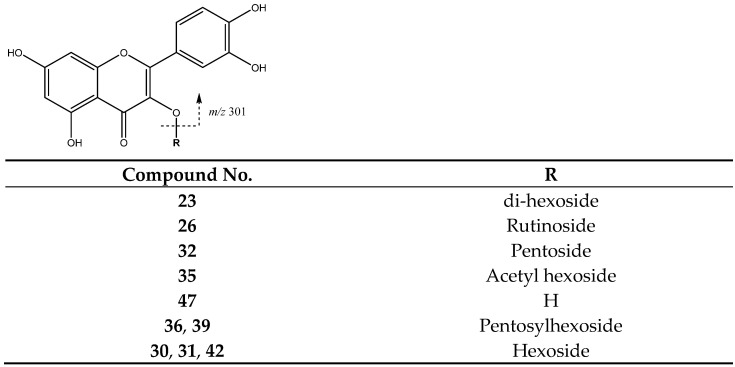
Flavonol glycosides structure and main fragments.

Peaks **30** (Rt = 23.78 min), **31** (Rt = 24.95 min) and **42** (Rt = 31.25 min) with [M-H]^−^ at *m*/*z* 463.0875 (C_21_H_19_O_12_) were tentatively identified as quercetin-hexoside isomers. These compounds suffer the loss of the hexoside [M-H-162]^−^ that results in the aglycone at *m*/*z* 301 (Figure 12). Meanwhile, peak **23** (Rt = 19.82 min) was tentatively assigned to quercetin di-hexoside with [M-H]^−^ at *m*/*z* 625.1378, showing that the loss of the two hexosides delivers the aglycone at *m*/*z* 301 [37].

Peak **32** (Rt = 25.35 min) was assigned to quercetin pentoside at [M-H]^−^ 433.0732 (C_20_H_17_O_11_), elucidated by the fragment of the aglycone due to loss of glycoside [38]. Peaks **36** (Rt = 27.77 min) and **39** (Rt = 29.24 min) had a [M-H]^−^ at *m*/*z* 595.1245 (C_26_H_27_O_16_), and were tentatively identified as quercetin-pentosyl-hexoside with the main fragment at *m*/*z* 301 due the loss of both glycoside units to yield the quercetin moiety. Peak **26** (Rt = 22.54 min) with [M-H]^−^ at *m*/*z* 609.1488 (C_27_H_29_O_16_) was identified as quercetin-rutinoside, showing the loss of the rutinoside moiety [M-H-308]^−^. Peak **35** (Rt = 29.09 min) with [M-H]^−^ at *m*/*z* 505.1025 (C_23_H_21_O_13_) was identified as quercetin-acetylhexoside with fragment at *m*/*z* 301 [M-162-H]^−^ due the loss of acetylhexoside moiety.

**Figure 13:**
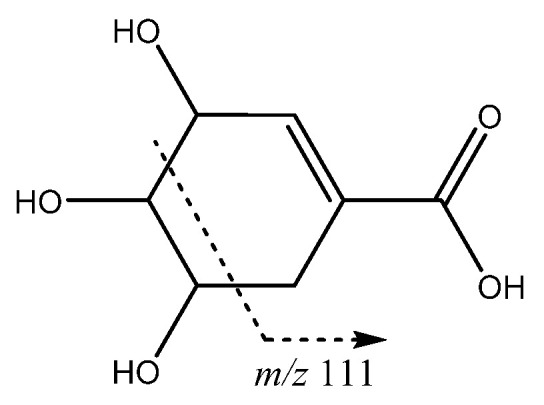
Shikimic acid structure and main fragments.

Phenolic acids and derivatives constitute another group of compounds found in these samples. The smallest acid found was peak **18** (Rt = 15.06 min) corresponding to shikimic acid (Figure 13). Main fragments at *m*/*z* 111 and 93 were generated from RDA fission and from subsequent loss of water, respectively.

**Figure 14:**
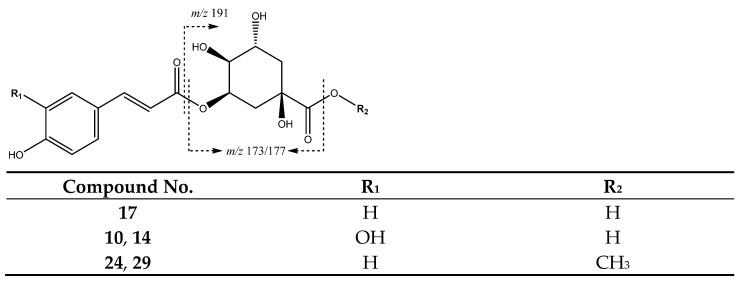
Structures and main fragments of coumaroyl and caffeic acid derivatives.

A series of 4-hydroxycinnamic acid derivatives were identified, as summarized in Table 2. For instance, as shown in Figure 14, peak **17** (Rt = 14.55 min) with [M-H]^−^ at *m*/*z* 337.0912 (C_16_H_17_O_8_) was assigned to a coumaroylquinic acid with a main fragment at *m*/*z* 173 due to the loss of water of the quinic acid ion [34].

**Figure 15:**
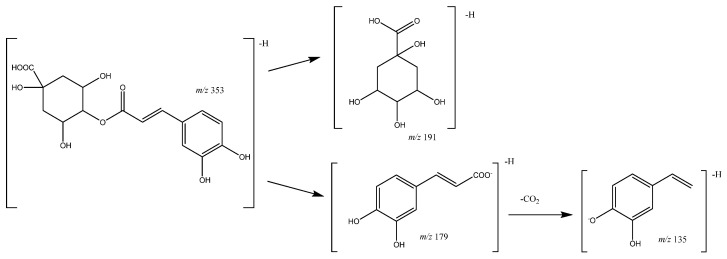
Caffeoylquinic acid fragmentation pathway.

In turn, peaks **24** (Rt = 20.82 min) and **29** (Rt = 23.60 min) correspond to coumaroylquinic acid methyl esters with [M-H]^−^ 351.1098 (C_17_H_19_O_8_), with a main fragment at *m*/*z* 177 due to the loss of CO from the quinic acid moiety. In addition, peaks **10** (Rt = 10.36 min) and **14** (Rt = 12.45 min) correspond to caffeoylquinic acids with a [M-H]^−^ at *m*/*z* 353.0809 (C_16_H_17_O_9_) and main fragments at *m*/*z* 191 [quinic acid-H]^−^, and 135 due to the loss of CO_2_ from the quinic acid ion, as shown in Figure 15 [39].

**Figure 16:**
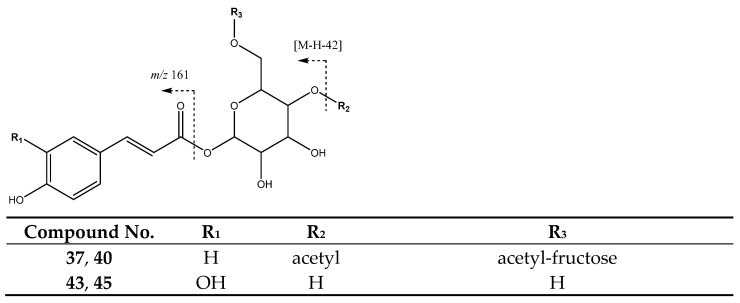
Structure and fragmentation of glycosylated and acetyl-glycosylated derivatives from hydroxycinnamic acids.

Peaks **43** (Rt = 31.45 min) and **45** (Rt = 34.65 min) show [M-H]^−^ at *m*/*z* 341.0872 (C_15_H_17_O_9_) and were tentatively identified as caffeoyl-hexoside isomers, which show a main fragment at *m*/*z* 161 [M-H-179]^−^ due to the loss of glycoside (Figure 16). Meanwhile, peaks **37** (Rt = 28.14 min) and **40** (Rt = 29.88 min), with [M-H]^−^ at 571.1675 (C_25_H_31_O_15_) were tentatively assigned to di-*O*-acetyl-*O*-*p*-coumaroylsucrose, with main fragments at *m*/*z* 553 [M-18-H]^−^ due to loss of water, at *m*/*z* 529 [M-42-H]^−^ because of acetyl loss, and at *m*/*z* 487 [M-84-H]^−^ due to the loss of both acetyl moieties [40].

**Figure 17:**
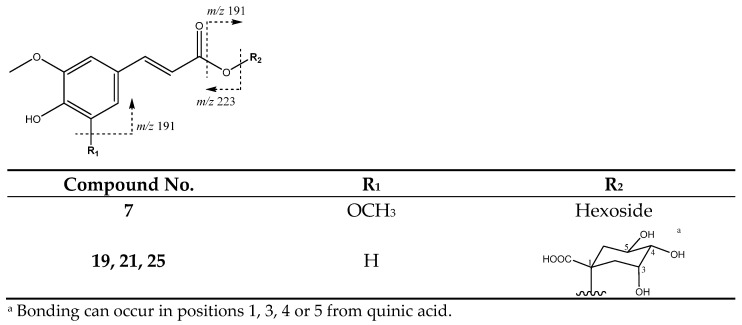
Structures and main fragments of sinapic acid and feruloyl quinic acid derivatives.

Additionally, as shown in Figure 17, peaks **19** (Rt = 15.75 min), **21** (Rt= 18.62 min) and **25** (Rt = 21.60 min), with [M-H]^−^ at 367.1012 (C_9_H_5_O_3_) were identified as feruloylquinic acid isomers, with main fragments at *m*/*z* 191 [quinic acid-H]^−^ and *m*/*z* 173 [quinic acid-H_2_O-H]^−^, as reported for these compounds [41]. Finally, peak **7** was assigned to sinapic acid hexoside showing [M−H]^−^ at *m*/*z* 385.1169 (C_17_H_21_O_10_) and main fragments at *m*/*z* 223 [M-H-162]^−^ due to the loss of the glycoside unit, at *m*/*z* 205 due the additional loss of water, and at *m*/*z* 191 due to the loss of the methoxy group from de aglycone.

Regarding the total number of polyphenols in *M. domestica* samples, the flesh of Jonagold shows the greatest number of compounds and exhibits the highest number of flavan-3-ols as well as being the most abundant in procyanidin tetramers and pentamers. Anna skins contain the second highest number of compounds and show flavonoids as the most abundant group of polyphenols.

When comparing data in the literature regarding compound characterization from apple skins, our results for Anna and Jonagold cultivars are similar to the total number of compounds and diversity in Golden Delicious and Braeburn cultivars from Slovenia [28]. In addition, both Costa Rican cultivars show a greater number and diversity in respect to other cultivars from Brazil and Canada [25,42,43]. In respect to flesh, the Jonagold cultivar is far superior to Anna and other cultivars from the literature, especially regarding proanthocyanidins both in total occurrence and in greater polymerization degree [24,25,44], for instance in procyanidin trimers, tetramers and pentamers found in Costa Rican Jonagold flesh.

In the case of glycosylated flavonoids, our findings show a similar number of compounds to European cultivars [24,28,44] and they indicate more diversity in quercetin derivatives. In respect to the occurrence of hydroxycinnamic acid derivatives, our results are within the range reported for cultivars from South Korea [45] and Europe [46,47,48]. Finally, for the chalcones group, our findings are similar to results reported for cultivars from Canada and China [25,49].

In sum, as within recent studies on other fruits [50], the profiling of polyphenols reveals high diversity in Costa Rican cultivars, which is in agreement with evidence showing that apples’ secondary metabolites profile is greatly influenced by location [44,51] as well as with findings from studies on other species indicating that tropical forests have a greater diversity of secondary metabolites [52]. Thus, the present work can be of interest for further research and future studies should take into consideration the parameters from the cultivars themselves, such as origin, location, soil composition and their relationship with chemical metabolites and bioactivities.

### 2.3. Antioxidant Activity

The DPPH and ORAC values obtained are summarized in Table 3. All samples show high antioxidant values, with Jonagold flesh presenting the best value with IC_50_ = 3.96 μg/mL for DPPH and 14.80 mmol Trolox equivalents/g for ORAC, followed by Anna and Jonagold skins, while Anna flesh exhibits the lowest antioxidant activity with IC_50_ = 11.33 μg/mL for DPPH and 4.53 mmol Trolox equivalents/g for ORAC. Regarding DPPH antioxidant findings from the literature, the results available for extracts show IC_50_ values for skins ranging between 41.41 and 55.54 µg/mL [53] and 710 µg/mL for flesh [48] in cultivars from India and Portugal, respectively, thus extracts from Anna and Jonagold cultivars show better results for both skins and flesh, as shown in Table 3. Another study on cultivars from Austria reported DPPH values ranging between 2.29 and 7.44 mmol TE/100 g DM [54] with our results for Anna and Jonagold cultivars showing values (2.12–8.05 mmol TE/100 g DM) within that range.

On the other hand, for ORAC, reports from the literature indicate values in the range of 8.60–44.07 mmol TE/100 g DM for skins and 2.4–42.97 mmol TE/100 g DM for apple flesh in cultivars from Germany [24] and Norway [47]. On the other hand, other studies report values varying between 0.45–10.62 mmol TE/100 g FW for skins and between 0.19 and 2.61 mmol TE/100 g FW for flesh in cultivars from Chile [56] and Italy [55]. Our results for skins (15.53–18.43 mmol TE/100 g DM and 3.10–4.23 mmol TE/100 g FW) and for flesh (6.37–20.99 TE/100 g DM and 1.01–2.94 mmol TE/100 g FW) from Anna and Jonagold cultivars fall within those ranges.

In addition, a correlation analysis was performed among the total phenolic contents (TPC, Table 1) and the antioxidant activity results from ORAC and DPPH methods. A significant negative correlation (*p* < 0.05) was found between TPC and DPPH results (R = −0.983) as wells as a significant positive correlation (*p* < 0.05) between TPC and ORAC values (R = 0.980). Therefore, these results align with previous findings reporting a correlation between total polyphenolic contents and different types of antioxidant activities [57].

Finally, the results for Jonagold flesh are of particular importance since there are few reports on the flesh being richer in polyphenols and having higher antioxidant activity than the skin, which points to the interest for further research on biological models. For instance, some studies have described the antioxidant mechanisms associated with proanthocyanidins with an increase in the Nuclear factor E2-related factor 2 (Nrf2) translocation to the nucleus [58], which activates the transcription of genes responsible for maintaining cellular redox homeostasis and protect cells from oxidative damage [59].

### 2.4. Cytotoxicity

**Figure 18:**
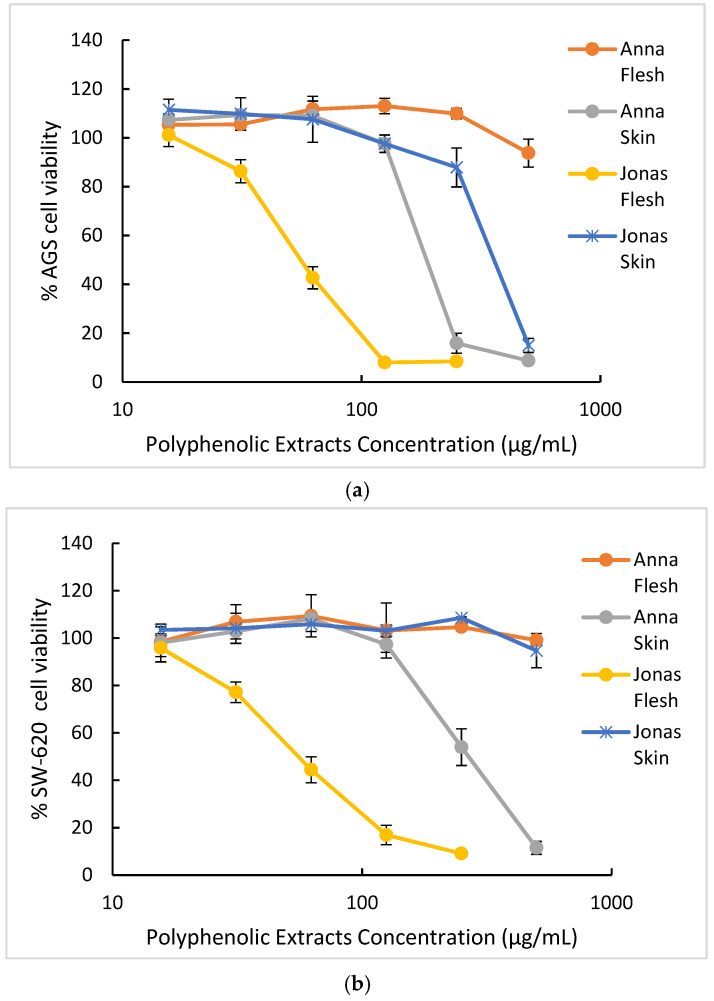
Cytotoxicity dose–response curves of apple extracts on AGS and SW620 tumor cell lines. Results are presented as mean ± SE of three independent experiments. (**a**) Samples in AGS cells (**b**) Samples in SW620 cells.

Table 4 summarizes the IC_50_ values for the cytotoxic effect of *M. domestica* extracts on different human carcinoma cells related to the digestive tract, namely AGS (gastric adenocarcinoma) and SW-620 (colorectal adenocarcinoma) cell lines, while the dose–response curves are displayed in Figure 18. The development of digestive tract cancers has been associated with lower consumption of vegetables and fruits [60]; in particular, 60% of stomach cancer and 43% of colon cancer are attributed to deficient consumption of vegetables [61]. In Costa Rica, colon cancer is the second most common cancer and gastric cancer has the third and fourth incidence rate in men and women, respectively [62]. Proanthocyanidins found in apples have been associated with exert antitumoral effects reaching and interacting directly with the gastrointestinal cells [63,64]. Thus, it is of interest to evaluate these extracts’ cytotoxicity using as targets these tumoral cancer cell lines.

As observed in Table 4, the best cytotoxic effects of Costa Rican *M. domestica* against AGS and SW-620 cells were observed for Jonagold cultivar, with the flesh sample (IC_50_ values of 60.0 ± 1.7 and 62.4 ± 5.2 μg/mL, respectively). Anna cultivar skin sample showed a moderate cytotoxic effect against AGS cells (IC_50_ of 167 ± 10 μg/mL).

The dose–response curves for each extract displayed in Figure 18 confirm Jonagold flesh as the best extract with the highest cytotoxic effect on both AGS and SW620 adenocarcinoma cell lines. In fact, both plots demonstrate a marked slope in the dose–response curves for this extract compared to the other samples. Anna and Jonagold skins show a more moderate cytotoxicity to obtain bioactive compounds, while Anna flesh represents the sample with the lowest cytotoxic effect in both tumoral cell lines tested.

Some studies have evaluated the cytotoxic effect of apples (*Malus domestica)* in tumoral cell lines and variations were observed for samples of the same species cultivated in different locations. Studies using French apples evaluated the cytotoxic effect against colorectal adenocarcinoma cells (SW-620) and esophageal adenocarcinoma (OE-33) showing 50% of cytotoxicity with similar concentrations of our study (45–60 μg/mL); however, for both studies, the extracts were enriched with a specific type of polyphenol [65,66]. Other studies showed moderate cytotoxic effect with acetone extracts from whole apple extracts from Lithuanian cultivars against the human colon adenocarcinoma cell line (HT-29) and human glioblastoma cell line (U-87), reporting an IC_50_ of 113.3 μg/mL and 119.7 μg/mL, respectively [67]. A lower cytotoxic effect was reported for acetone and alcoholic extracts from apple (*M. domestica*) pomace cultivated in India. These Indian apples achieved 50% cytotoxicity only in oral carcinoma (KB) with concentrations of 100 μg/mL, but for cervical squamous cells carcinoma (SiHa) and colorectal adenocarcinoma (HT-29), 50% was not reached even with treatment of 400 μg/mL [68]. On the other hand, studies of Indian *M. domestica* apples achieved an improved cytotoxic effect using innovative delivering strategies such as silver nanoparticles. This approach permits an IC_50_ of 10 μg/mL [69] and 33.8 μg/mL [70] to be achieved against breast cancer cells (MFC-7).

The cytotoxic effect in tumor cell lines has been reported for other *Malus* species, also demonstrating very fluctuating results. *Malus sieversii* acetone extracts, grown in China, were assessed on breast cancer cell lines (MCF-7 and MAD-MB-231) and showed a very low cytotoxic effect (IC_50_ of 33.44 mg/mL and 20.94 mg/mL) [71]. Similarly, a methanolic extract of Chinese apples, *Malus pumila*, were evaluated against cancer colon cells (SW-480), stomach cancer cells (BCG 803) and esophageal cancer cells (CaEs-12) and a weak cytotoxic activity was reported with IC_50_ varying between 3.5–4.3 mg/mL in all cell lines [72]. In the opposite side, the cytotoxicity of Chinese apples, *M. pumila*, was evaluated against liver hepatocellular carcinoma (HepG2) and a strong inhibitory grown rate of 50% was achieved with concentration of less than 4 μg/mL for pulp extracts and less than 20 μg/mL for skin extracts [73]. Finally, ornamental crabapple *Malus* sp. (“red splendor”) has also been studied, and the cytotoxic activity showed values of 48.3 μg/mL, 64.5 μg/mL and 78.9 μg/mL, for SW-480, BCG 803 and CaEs-17, respectively [72].

In addition to the IC_50_ values used to quantify the cytotoxic effect, Table 4 shows the selectivity index, which is defined as the ratio of IC_50_ values of non-tumor cells to cancer cells. The highest selectivity index values in this study correspond to the Jonagold flesh sample (5.1 for AGS cells and 4.9 for SW-620 cells) and the Anna skin sample (3.0 for AGS cells). According to previous reports, extracts with SI greater than three are considered to have high selectivity towards cancer cells and suggest a possible therapeutic potential [74,75]. For *M. domestica* extracts, a comparison of IC_50_ values of non-tumor cells to cancer cells has been reported previously for breast cancer cell lines. The selectivity ratio in this Indian apple was 2.2, which is a lower value compared to our study, even though the Indian apple extracts were applied to the cells using nanoparticle delivery systems [70].

In addition, correlation analysis was performed between the cytotoxicity results obtained and total phenolic contents. Significant negative correlation (*p* < 0.05) was found between IC_50_ cytotoxic values on SW620 cancer cells and TPC (*r* = −0.908) and between IC_50_ cytotoxic values on AGS cancer cells and TPC (*r* = −0.902). Furthermore, correlation analysis performed between the IC_50_ cytotoxic activity in both adenocarcinoma cell lines (Table 4) and the number of compounds identified for each polyphenol group (Table 2) showed no significant correlation (*p* < 0.05) with HCA, chalcones or flavonoids for either SW620 or AGS cell lines. In contrast, a significant negative correlation (*p* < 0.05) with the number of procyanidins was found for cytotoxicity results on both SW620 (*r* = −0.978) and AGS (*r* = −0.894) cell lines. These r coefficients represent similar and higher values, respectively, than the ones for TPC, suggesting procyanidin’s major contribution to the cytotoxic activity against both tumor cells.

The predominant role of proanthocyanidins in the cytotoxic effect against tumoral cells has been widely documented for grape seeds extracts [76,77,78,79]. However, not many reports are available for other natural sources; some of the few reports include exotic fruits such as Japanese Quince [80] and *Bactris guineensis* [81] and other widespread consumed fruits, such as berries [82].

The association of proanthocyanidins and cytotoxic effect in tumoral cells has been linked to the degree of polymerization of these polyphenols. For grapes, grape seeds and pine bark assays in colon cancer cells (HCT116, SW-480, SW-620, HT-29, Caco-2, RKO and LoVo), the anti-proliferative effect positively correlated with an increase in the degree of polymerization [83]. Another report [65] compared two polyphenol-enriched fractions from *M. domestica*, reporting a 50% inhibition of colorectal carcinoma (SW-620) cell growth with 45 μg/mL of the fraction rich in polymers and no effect in the monomer fraction even at a concentration of 100 μg/mL. Other studies in esophageal gastric adenocarcinoma demonstrated that oligomer procyanidins showed more potent antiproliferative activities that the monomeric and dimeric procyanidins [66]. These reports are consistent with the pattern shown in the present study. The strongest cytotoxic activity, an IC_50_ of 60 and 63 μg/mL in AGS and SW-620, respectively, was assessed for flesh samples of the Jonagold cultivar (Table 4) which is the one showing an enriched profile of proanthocyanidins oligomers (Table 2), specifically trimers, tetramers and pentamers B-type procyanidins.

The antitumor effect of proanthocyanidins has been associated with an apoptotic induction and a regulation of inflammatory pathways that ends in an inhibition of the tumor cell proliferation [81,84,85]. Some reports from grape seeds and apple procyanidins (*M. pumila*) have described an induction of cell cycle arrest by down-regulation of cyclin D1, CDK4 and survivin. In addition, these reports describe an induction of apoptosis through an increase in mitochondrial membrane permeability, a cytochrome c release and enhance of caspase 3 and caspase 9 expression and activation, which represents a hallmark of apoptosis [76,85,86]. However, despite these preliminary reports, the specific mechanism has yet to be elucidated. Reports on the bioavailability of procyanidins indicate that these molecules reach the colon almost intact and would interact there with colorectal cancer cells [64] similar to the ones evaluated in this work. In sum, the promising results obtained for Jonagold flesh suggest that there is a need for further studies, for instance in other cancer cell lines, to determine the prospective of these Costa Rican apples as source of enriched proanthocyanidins extracts and their related bioactivities.

## 3. Materials and Methods

### 3.1. Materials, Reagents and Solvents

*M. domestica* fruits of Anna and Jonagold cultivars were acquired in ripe state in late summer from producers in Los Santos, Costa Rica. Cultivars were confirmed with the support of the Costa Rican National Herbarium and vouchers are deposited there. Reagents, such as fluorescein, 2,2-azobis(2-amidinopropane) dihydrochloride (AAPH), 2,2-diphenyl-1-picrylhidrazyl (DPPH), Trolox, gallic acid, and Amberlite XAD-7 resin, fetal bovine serum, glutamine, penicillin, streptomycin, amphotericin B, trypsin–EDTA, were provided by Sigma-Aldrich (St. Louis, MO, USA). Human gastric adenocarcinoma cell line AGS, human colorectal adenocarcinoma SW 620 and monkey normal epithelial kidney cells Vero were obtained from American Type Culture Collection (ATCC, Rockville, MD, USA), while solvents such as acetone, chloroform and methanol were purchased from Baker (Center Valley, PA, USA), while DMSO was acquired from Sigma-Aldrich (St. Louis, MO, USA).

### 3.2. Phenolic Extracts from Malus Domestica Fruits

*M. domestica* fruits were rinsed in water, peeled, and both skin and flesh material were frozen at −20 °C and then lyophilized in a Free Zone Cascade Benchtop Freeze Dry System 720401000 (Labconco, Kansas, MO, USA), with Ice Holding Capacity of 4.5 L and Collector Temperature of −105 °C and system vacuum level < 133 × 10^−3^ mbar. The lyophilized material was preserved at −20 °C until extraction. Freeze-dried samples were extracted under Pressurized Liquid Extraction (PLE) conditions, in a Dionex™ ASE™ 150 Accelerated Solvent Extractor (Thermo Scientific™, Walthman, MA, USA) using methanol:water (70:30) as solvent in a 34 mL cell, at 40 °C. Next, the extract was evaporated under vacuum to eliminate the methanol and the aqueous phase was washed with ethyl acetate and chloroform to remove less polar compounds. Afterwards, the aqueous extract was evaporated under vacuum to eliminate organic solvent residues and was eluted (2 mL/min) in Amberlite XAD7 column (150 mm × 20 mm), starting with 300 mL of water to remove sugars, and then with 200 mL each of methanol:water (80:20) and pure methanol to obtain the polyphenols. Finally, the enriched extract was obtained after evaporating to dryness at 40 °C using a Buchi™ 215 (Flawil, Switzerland) rotavapor.

### 3.3. Total Phenolic Content

The polyphenolic content was determined as previously reported [87] by a modification of the Folin–Ciocalteu (FC) method [88], whose reagent is composed of a mixture of phosphotungstic and phosphomolybdic acids. Each sample was dissolved in MeOH (0.1% HCl) and combined with 0.5 mL of FC reagent. Afterwards, 10 mL of Na_2_CO_3_ (7.5%) were added and the volume was completed to 25 mL with water. Blanks were prepared in a similar way, but using 0.5 mL of MeOH (0.1% HCl) instead of the sample. The mixture was left standing in the dark for 1 h and then the absorbance was measured at 750 nm. Values obtained were extrapolated in a gallic acid calibration curve. Total phenolic content was expressed as mg gallic acid equivalents (GAE)/g sample. Analyses were performed in triplicate.

### 3.4. UPLC-ESI-MS Analysis

The UPLC-MS system used to analyze the composition of *M. domestica* extracts consisted of a Xevo G2-XS QTOF (Waters, UK) coupled with an AQUITY H Class UPLC system with quaternary pump. ESI source parameters were set to a capillary voltage of 2 kV, sampling cone of 20 eV, source temperature of 150 °C, and source offset of 10 °C. The desolvation temperature was set at 450 °C, the cone gas flow at 0 L/h and the desolvation gas flow at 900 L/h.

Measurement was performed in MS^e^ high resolution negative mode using an acquisition mass range from 100 *m*/*z* to 2000 *m*/*z* and a scan rate of 0.5 s, where fragmentation was carried out using Independent Data Acquisition for all eluting compounds with collision energy ramp from 20 V to 30 V storing at the high energy function. Instrument calibration was applied in the mass range of the measurement with sodium formate. Lock mass correction was applied directly to the measurement using leucine enkephalin infusion measured each 30 s during the run. The data was analyzed using MassLynx V4.2 software from Waters.

Separation was carried out on a Luna RP-C18 column (150 mm × 4.6 mm i.d. × 4 µm, Phenomenex, Torrance, CA, USA) with a pre-column filter (Phenomenex, Torrance, CA, USA). Solvents used in the mobile phase were water with 0.1% formic acid (A), methanol with 0,1% formic acid (B) and acetonitrile with 0.1% formic acid (C). Then, 5 μL of sample was injected with a flow rate of 0.5 mL/min at 40 °C. The chromatographic gradient started at 83% A, 12% B and 7% C, changing to 79.2% A, 12% B and 8.8% C at 4.8 min, then to 74% A, 15% B, and 11% C at 14.8 min, then to 0% A, 85% B and 15% C at 48 min, holding it for 10 min. Then, the column was equilibrated for 5 min to initial conditions.

### 3.5. DPPH Radical-Scavenging Activity

DPPH evaluation was performed as previously reported [89] and was expressed as IC_50_ (µg/mL), which is the amount of sample required to reach the 50% radical-scavenging activity, and also as mmol of Trolox equivalents (TE)/g extract. Briefly, a solution of 2,2-diphenyl-1-picrylhidrazyl (DPPH) (0.25 mM) was prepared using methanol as solvent. Next, 0.5 mL of this solution was mixed with 1 mL of extract or Trolox at different concentrations, and incubated at 25 °C in the dark for 30 min. DPPH absorbance was measured at 517 nm. Blanks were prepared for each concentration. The percentage of the radical-scavenging activity of the sample or Trolox was plotted against its concentration to calculate IC_50_ (µg/mL). The samples were analyzed in three independent assays. In order to express the DPPH results as mmol TE/g extract, the IC_50_ (µg/mL) of Trolox was converted to mmol/mL using Trolox molecular weight (250.29 mg/mmol) and then dividing by the IC_50_ of each sample.

### 3.6. ORAC Antioxidant Activity

The Oxygen Radical Absorbance Capacity (ORAC) antioxidant activity was determined following a method previously described [90] using fluorescein as a fluorescence probe. The reaction was performed in 75 mM phosphate buffer (pH 7.4) at 37 °C. The final assay mixture consisted of AAPH (12 mM), fluorescein (70 nM), and either Trolox (1–8 µM) or the extract at different concentrations. Fluorescence was recorded every minute for 98 min in black 96-well untreated microplates (Nunc, Denmark), using a Polarstar Galaxy plate reader (BMG Labtechnologies GmbH, Offenburg, Germany) with 485-P excitation and 520-P emission filters. Fluostar Galaxy software version 4.11-0 (BMG Labtechnologies GmbH, Offenburg, Germany) was used to measure fluorescence. Fluorescein was diluted from a stock solution (1.17 mM) in 75 mM phosphate buffer (pH 7.4), while AAPH and Trolox solutions were freshly prepared. All reaction mixtures were prepared in duplicate and three independent runs were completed for each extract. Fluorescence measurements were normalized to the curve of the blank (no antioxidant). From the normalized curves, the area under the fluorescence decay curve (AUC) was calculated as:

(1) $$\mathrm{AUC}=1+\overset{i=98}{\underset{i=1}{\sum }}f_{i}/f_{0}$$

where *f*_0_ is the initial fluorescence reading at 0 min and *f_i_* is the fluorescence reading at time *i*. The net AUC corresponding to a sample was calculated as follows:

Net AUC = AUC_antioxidant_ − AUC_blank_ (2)

The regression equation between net AUC and antioxidant concentration was calculated. The ORAC value was estimated by dividing the slope of the latter equation by the slope of the Trolox line obtained for the same assay. Final ORAC values were expressed as mmol of Trolox equivalents (TE)/g of phenolic extract.

### 3.7. Evaluation of Cytotoxicity of Extracts

#### 3.7.1. Cell Culture

The human gastric adenocarcinoma cell line AGS, the human colorectal adenocarcinoma SW 620 and monkey normal epithelial kidney cells Vero were grown in minimum essential Eagle’s medium (MEM) containing 10% fetal bovine serum (FBS) in the presence of 2 mmol/L glutamine, 100 IUmL^−1^ penicillin, 100 μg/mL streptomycin and 0.25 μg/mL amphotericin B. The cells were grown in a humidified atmosphere containing 5% CO_2_ at 37 °C and were sub-cultured by detaching with trypsin–EDTA solution at about 70–80% confluence. For the experiments, 100 μL of a cell suspension of 1.5 × 10^5^ cells/mL were seeded overnight into 96-well plates. The cells were further exposed for 48 h to various concentrations of extracts (50 μL), dissolved in DMSO and diluted with cell culture medium to final concentrations between 15–500 μg/mL. The DMSO concentrations used in the experiments were below of 0.1% (*v*/*v*) and control cultures were prepared with the addition of DMSO (vehicle control).

#### 3.7.2. Assessment of Cytotoxicity by MTT Assay

After incubation for 48 h, MTT assays were performed to evaluate the cell viability. The decrease in the viability correlates with the cytotoxic activity of the extract. Briefly, the medium was eliminated, cells were washed twice with 100 μL of PBS and incubated with 100 μL MTT solution (3-(4,5-dimethylthiazolyl-2)-2,5-diphenyltetrazolium bromide, 5 mg/mL in cell culture medium) for 2 h at 37 °C. The formazan crystals formed were dissolved in 100 μL of ethanol 95% and the absorbance was read at 570 nm in a microplate reader. Dose–response curves were established for each extract and the concentration, which is enough to reduce the cell viability by 50% (IC_50_), was calculated.

In order to evaluate whether the cytotoxicity activity was specific against the cancer cells, a selectivity index (SI) was determined. This index is defined as the ratio of IC_50_ values of normal epithelial kidney cells (Vero) to cancer cells (AGS or SW620).

### 3.8. Statistical Analysis

One-way analysis of variance (ANOVA) followed by Tukey’s post hoc test was applied to TPC, DPPH, ORAC and cytotoxicity results, and differences were considered significant at *p* < 0.05. In order to evaluate whether the total phenolic contents (TPC) contributes to the antioxidant activity evaluated with DPPH and ORAC methodologies, a correlation analysis was carried out as well as cytotoxicity assays. R (version x64 4.1.1) was used as the statistical program.

## 4. Conclusions

The UPLC-HRMS analysis using the QTOF-ESI MS technique allowed 106 compounds to be characterized in phenolic enriched extracts of skins and flesh of Anna and Jonagold apple cultivars in Costa Rica. Among them, the flesh of the Jonagold cultivar displayed the most abundant number of polyphenols and also exhibited higher and more diversified procyanidin oligomers than cultivars from other countries reported in the literature. Furthermore, this extract also showed the best results for TPC, ORAC and DPPH antioxidant activities as well as for cytotoxicity IC_50_ values against SW620 and AGS cancer cell lines. In addition, the abundance of procyanidins showed a significant positive correlation (*p* < 0.05) with the ORAC results and a significant negative correlation (*p* < 0.05) with DPPH and cytotoxicity towards AGS and SW620 tumor. These findings align with the fact that procyanidin oligomers were more abundant and presented a higher degree of polymerization, including tetramers and pentamers, in the flesh of Jonagold extract, displaying better bioactivity effects. The overall results from this study and particularly the ones obtained for the flesh of Jonagold cultivar, support findings suggesting the importance of considering fruit varieties [91]. As mentioned, the higher degree of polymerization in procyanidins has been linked with anti-inflammatory and anticancer activities [92], therefore additional research would contribute to determining the potential health benefits of these extracts.

## Figures and Tables

**Table 1:** Total phenolic content from the extracts of *M. domestica* cultivars.

Sample	Lyophilization Yield (%) ^1,2^	Extraction Yield (mg Extract/g DM) ^2,3^	Total Phenolic Content (mg GAE/g Extract) ^2,4^
*Anna*			
Skin	22.95 ± 0.21	16.47 ± 0.62	472.26 ^a^ ± 5.5
Flesh	15.92 ± 0.45	10.69 ± 0.34	354.46 ^b^ ± 8.4
*Jonagold*			
Skin	19.94 ± 0.68	20.87 ± 0.55	417.07 ^c^ ± 12.3
Flesh	14.03 ± 0.53	14.18 ± 0.84	608.78 ^d^ ± 4.4

^1^ g of dry material (DM)/g of fresh weight (FW) expressed as %. ^2^ Values represent average ± standard deviation (S.D.) from three independent runs for each sample (*n* = 3). ^3^ mg of extract/g of dry material. ^4^ Different superscript letters indicate that differences are significant at *p* < 0.05 using ANOVA with a Tukey post hoc test.

**Table 2:** Profile of the phenolic compounds identified by UPLC-DAD-ESI-MS/MS in Costa Rican apple cultivars ^1^.

No	Tentative Identification	Rt (min)	[M-H]^−^	Formula	MS2 Fragments	Sample ^1^
	*Hydroxycinamic acids*					
**7**	Sinapic acid hexoside	8.91	385.1169	C_17_H_22_O_10_	[385]: 205, 223	JF
**10**	Caffeoylquinic acid (I of II)	10.36	353.0871	C_16_H_17_O_9_	[353]: 191, 179	JF
**14**	Caffeoylquinic acid (II of II)	12.45	353.0809	C_16_H_17_O_9_	[353]: 191, 180	JF
**17**	*p*-Coumaroylquinic acid	14.55	337.0912	C_16_H_18_O_8_	[337]: 173	AF, JF
**18**	Shikimic acid	15.06	173.0447	C_7_H_9_O_5_	[173]: 93, 111	AF
**19**	Feruloylquinic acid (I of III)	15.75	367.0983	C_17_H_20_O_9_	[367]: 173, 191	AS, AF
**21**	Feruloylquinic acid (II of III)	18.62	367.1012	C_17_H_20_O_9_	[367]: 173, 191	AS, AF, JS, JF
**24**	Methyl-*p*-coumaroylquinic acid (I of II)	20.82	351.1098	C_17_H_20_O_9_	[351]: 177	AS, AF
**25**	Feruloylquinic acid (III of III)	21.60	367.0983	C_17_H_20_O_9_	[367]: 173, 191	AS, AF, JF
**29**	Methyl-*p*-coumaroylquinic acid (II of II)	23.60	351.1098	C_17_H_19_O_8_	[351]: 177	AF
**37**	Di-*O*-acetyl-*O*-*p*-coumaroylsucrose (I of II)	28.14	571.1675	C_25_H_31_O_15_	[571]: 529, 553	JF
**40**	Di-*O*-acetyl-*O*-*p*-coumaroylsucrose (II of II)	29.88	571.1673	C_25_H_31_O_15_	[571]: 529, 554	JS, JF
**43**	Caffeoyl hexoside (I of II)	31.45	341.084	C_15_H_17_O_9_	[341]: 161, 179	AS, AF, JS, JF
**45**	Caffeoyl hexoside (II of II)	34.65	341.084	C_15_H_17_O_9_	[341]: 161, 179	AS, AF, JS
	*Chalcones*					
**33**	Phloretin-pentosilhexoside (I of II)	26.14	567.1725	C_26_H_31_O_14_	[567]: 273	AS, AF, JS, JF
**34**	Phloretin-pentosilhexoside (II of II)	26.95	567.1725	C_26_H_31_O_14_	[567]: 273	JF
**41**	Phloridzin	30.00	435.1312	C_21_H_24_O_10_	[435]: 167, 273	AS, AF, JS, JF
**48**	Phloretin	40.98	273.0757	C_15_H_13_O_5_	[273]: 167, 201	AS, AF, JS
	*Flavonoids*					
**23**	Quercetin di-hexoside	19.82	625.1378	C_30_H_25_O_13_	[625]: 300, 301	AS, JS, JF
**26**	Quercetin-rutinoside	22.54	609.1459	C_27_H_29_O_16_	[609]:300, 301	AS, JS, JF
**30**	Quercetin-hexoside (I of III)	23.78	463.0878	C_21_H_19_O_12_	[463]: 300, 301	AS, JS, JF
**31**	Quercetin-hexoside (II of III)	24.95	463.0878	C_21_H_19_O_12_	[463]: 300, 301	AS, AF
**32**	Quercetin-pentoside	25.35	433.0732	C_20_H_17_O_11_	[433]: 300, 301	AS, AF, JF
**35**	Quercetin acetyl hexoside	27.16	505.1002	C_23_H_22_O_13_	[505]: 300, 301	AS, JS, JF
**36**	Quercetin-pentosylhexoside	27.77	595.1245	C_26_H_27_O_16_	[595]: 300, 301	JF
**38**	Kaempferol-hexoside	28.63	447.0928	C_21_H_19_O_11_	[447]: 284.,285	AS, AF, JS, JF
**39**	Quercetin pentosyl hexoside	29.24	587.1043	C_25_H_26_O_15_	[595]: 300, 301	JS, JF
**42**	Quercetin-hexoside (III of III)	31.25	463.0834	C_21_H_20_O_12_	[463]: 300, 301	AS, AF, JS, JF
**46**	Kaempferol-hexoside	36.18	477.0944	C_21_H_20_O_11_	[447]: 284, 285	AS, AF, JS
**47**	Quercetin	37.19	301.0353	C_15_H_10_O_7_	[301]: 151, 179, 255, 273, 283	AS, JS
	*Proantocyanidins*					
**1**	Procyanidin B-type dimer (I of IV)	5.74	577.1292	C_30_H_25_O_12_	[577]: 287, 289, 407, 425, 451, 559	JS, JF
**2**	Procyanidin B-type dimer (II of IV)	6.12	577.1294	C_30_H_25_O_12_	[577]: 287, 289, 407, 425, 451, 559	JF
**3**	Procyanidin B-type dimer (III of IV)	6.57	577.1343	C_30_H_25_O_12_	[577]: 287, 289, 407, 425, 451, 559	AS, AF, JF
**4**	Procyanidin B-type trimer (I of III)	7.31	865.2004	C_45_H_37_O_18_	[865]: 287, 289, 575, 577, 695, 713, 739	JF
**5**	Procyanidin A-type dimer	7.67	591.1147	C_30_H_24_O_13_	[575]: 289, 449	AF, JS
**6**	Procyanidin B-type trimer (II of III)	8.5	865.2004	C_45_H_37_O_18_	[865]: 287, 289, 575, 577, 695, 713, 739	AS, JF
**8**	Catechin	9.01	289.0708	C_15_H_14_O_6_	[289]: 205,245, 271	AS, JF
**9**	Procyanidin B-type dimer (IV of IV)	9.52	577.1392	C_30_H_25_O_12_	[577]: 287, 289, 407, 425, 451, 559	AS, AF, JF
**11**	Procyanidin B-type trimer (III of III)	11.48	865.2004	C_45_H_37_O_18_	[865]: 287, 289, 575, 577, 695, 713, 739	AS, JF
**12**	Procyanidin tetramer B (I of III)	11.76	1153.2629	C_60_H_49_O_24_	[1153]: 287, 289, 575, 577, 863, 865, 983, 1001, 1027, 1135	JF
**13**	Epicatechin	12.14	289.0708	C_15_H_13_O_6_	[289]: 205, 245, 271	AS, JF
**15**	Procyanidin B-type tetramer (II of III)	12.82	1153.2704	C_60_H_49_O_24_	[1153]: 287, 289, 575, 577, 863, 865, 983, 1001, 1027, 1135	JF
**20**	Procyanidin B-type pentamer (I of III)	16.55	1441.2936	C_75_H_61_O_30_	[1441]: 287, 289, 575, 577, 865, 1153, 1315	JF
**22**	Procyanidin B-type pentamer (II of III)	19.13	1441.2939	C_75_H_61_O_30_	[1441]: 287, 289, 575, 577, 865, 1153, 1315	JS, JF
**27**	(epi)catechin 3-*O*-gallate	22.89	609.1459	C_27_H_29_O_16_	[441]: 153, 289, 315	JF
**28**	Procyanidin B-type tetramer (III of III)	23.20	1153.2701	C_60_H_49_O_24_	[1153]: 287, 289, 575, 577, 863, 865, 983, 1001, 1027, 1135	AS, JS, JF
**44**	Procyanidin B-type pentamer (III of III)	32.05	1441.2931	C_75_H_61_O_30_	[1441]: 287, 289, 575, 577, 865, 1153, 1315	JF
	*Others*					
**16**	Vomifoliol-pentosilhexoside	13.97	517.2293	C_24_H_37_O_12_	[517]: 205, 385	AS, JS, JF

^1^ Anna skin (AS), Anna flesh (AF), Jonagold skin (JS), Jonagold flesh (JF).

**Table 3:** DPPH and ORAC antioxidant activity from the extracts of *M. domestica* cultivars.

Sample	DPPH ^1,2^		ORAC ^1.2^
	IC_50_ (μg Extract/mL)	(mmol TE/g Extract)	(mmol TE/g Extract)
*Anna*			
Skin	6.90 ^a^ ± 0.02	3.25 ^a^ ± 0.01	11.19 ^a^ ± 0.25
Flesh	11.33 ^b^ ± 0.05	1.98 ^b^ ± 0.01	5.96 ^b^ ± 0.23
*Jonagold*			
Skin	9.76 ^c^ ± 0.17	2.30 ^c^ ± 0.04	7.44 ^c^ ± 0.10
Flesh	3.96 ^d^ ± 0.02	5.68 ^d^ ± 0.03	14.80 ^d^ ± 0.26

^1^ Values are expressed as mean ± S.D. ^2^ Different superscript letters in the same column indicate that differences are significant at *p* < 0.05 using ANOVA with a Tukey post hoc as statistical test. ORAC, oxygen radical absorbance capacity; DPPH, 2,2-diphenyl-1-picrylhidrazyl method.

**Table 4:** Cytotoxicity of *M. domestica* extracts to gastric (AGS) and colon (SW-620) carcinoma cells as well as to Vero non-tumoral cells.

Sample	IC_50_ (µg/mL) ^1,2^ (SI) ^3^		
	AGS	SW-620	Vero
*Anna*			
Skin	167.22 ^a,^* ± 10 (3.0)	295.93 ^b,#^ ± 29 (1.7)	> 500 ^a,≠^
Flesh	> 500 ^b,^*	> 500 ^a,*^	> 500 ^a,^*
*Jonagold*			
Skin	398.44 ^c,^* ± 7 (1.3)	> 500 ^a,^*	305.72^.b,^* ± 30
Flesh	60.03 ^d,^* ± 1.7 (5.1)	62.41 ^c,^* ± 5.2 (4.9)	> 500 ^a,#^

^1^ Different superscript letter in the same column indicates that differences are significant at *p* < 0.05 using ANOVA with a Tukey post hoc as statistical test. ^2^ Different superscript signs in the same row indicate that differences are significant at *p* < 0.05 using ANOVA with a Tukey post hoc as statistical test. ^3^ Selectivity Index.

## Data Availability

The data presented in this study are available within this article.

## Sample Availability

Samples of the compounds are not available from the authors.
